# Proteasomal Degradation Machinery: Favorite Target of HIV-1 Proteins

**DOI:** 10.3389/fmicb.2018.02738

**Published:** 2018-11-21

**Authors:** Sneh Lata, Ritu Mishra, Akhil C. Banerjea

**Affiliations:** Virology Lab II, National Institute of Immunology, New Delhi, India

**Keywords:** proteasome, proteasome 20S, ubiquitination, deubiquitinase (DUB), HIV-1, Tat, quinone oxidoreductase 1 (NQO1)

## Abstract

Proteasomal degradation pathways play a central role in regulating a variety of protein functions by controlling not only their turnover but also the physiological behavior of the cell. This makes it an attractive target for the pathogens, especially viruses which rely on the host cellular machinery for their propagation and pathogenesis. Viruses have evolutionarily developed various strategies to manipulate the host proteasomal machinery thereby creating a cellular environment favorable for their own survival and replication. Human immunodeficiency virus-1 (HIV-1) is one of the most dreadful viruses which has rapidly spread throughout the world and caused high mortality due to its high evolution rate. Here, we review the various mechanisms adopted by HIV-1 to exploit the cellular proteasomal machinery in order to escape the host restriction factors and components of host immune system for supporting its own multiplication, and successfully created an infection.

## Introduction

The proteasomal machinery, which is present in all eukaryotes, is responsible for the degradation of damaged, misfolded, or unfolded proteins by proteolysis in the cell. Being an essential basic intracellular phenomenon, proteasomal machinery is known to influence almost all the cellular processes including signaling pathways, chromatin structure, endocytosis, apoptosis, neuronal function, development, and immunity (Evans, 2005). Proteasome is the principle component of this machinery which carries out the proteolysis with the help of proteases. Proteasomes are located in both the nucleus and cytoplasm of the cell (Peters et al., 1994). There are two pathways of proteasomal degradation of proteins, and they are listed below.

### Ubiquitin-Dependent Proteasomal Degradation Pathway

**FIGURE 1:**
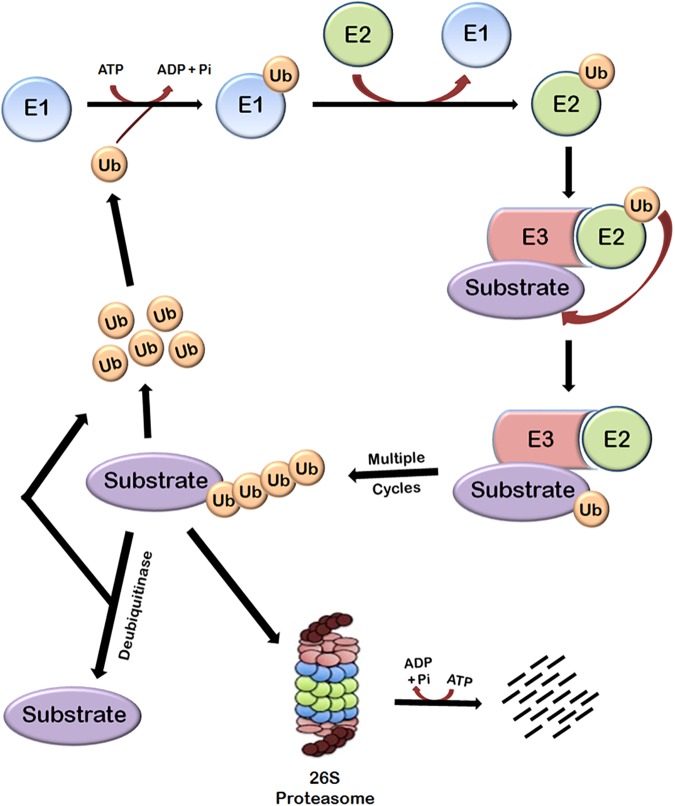
Ubiquitin-dependent proteasomal degradation pathway. The cellular proteins are targeted to ubiquitin-dependent degradation by a set of three enzymes. E1 first binds to ubiquitin molecule and transfers it to E2. E2 interacts with substrate bund E3 and ubiquitin is transferred to the substrate. Repeated actions of these three enzymes cause polyubiquitination of the substrate followed by its degradation by proteasome and release of free ubiquitin molecules which can again bind with E1 to initiate the ubiquitination of another substrate. This process requires energy which is obtained from the hydrolysis of ATP. Polyubiquitinated substrates can also be deubiquitinated by another class of enzymes called deubiquitinases, which results in the release of free substrate molecules.

Protein turnover in cells is principally regulated by ubiquitin-proteasome system (UPS). This system is basically composed of three main components—the proteasome holoenzymes, various ubiquitin ligases, and multiple de-ubiquitinating enzymes (DUBs) (Bailey-Elkin et al., 2017). Ubiquitin, which is a small protein of 76 amino acids, is linked to the proteins that are targeted for degradation through a multistep process called ubiquitination that is carried out by several enzymes. Ubiquitination involves the covalent modification of a lysine residue of the proteins targeted for degradation which is mediated by the action of three enzymes. In the first step, E1 (ubiquitin activating enzyme) binds to the ubiquitin molecule through its active site. This step involves the hydrolysis of one ATP molecule. In the next step, ubiquitin is transferred to the active site of E2 (ubiquitin conjugating enzyme). In the third step, E3 (ubiquitin ligase enzyme) recognizes the substrate protein to be degraded and catalyzes the transfer of ubiquitin molecule from E2 to the substrate. These three steps are repeated several times resulting in the formation of polyubiquitin chains (Figure 1).

Substrate proteins can also remain mono-ubiquitinated which broadly earmarks the substrate toward a particular function (Weake and Workman, 2008). There are seven lysine residues in ubiquitin molecules through which they can be ligated to each other. Polyubiquitin chains in which ubiquitin molecules are interlinked at lysine 48 residue target the substrate to proteasomal degradation and other types of chains have different roles (Pickart and Fushman, 2004; Xu et al., 2009). Polyubiquitinated substrate is moved to 26S proteasome and degraded. This also requires hydrolysis of ATP because energy is required for unfolding the protein (Smith et al., 2005). The linking of Ub can be reversed through the isopeptidase activities of deubiquitinases (DUBs), a phenomenon known as deubiquitination (Nijman et al., 2005; Lindner, 2007), which will be discussed later.

#### Exploitation of 26S Proteasome by HIV-1

**FIGURE 2:**
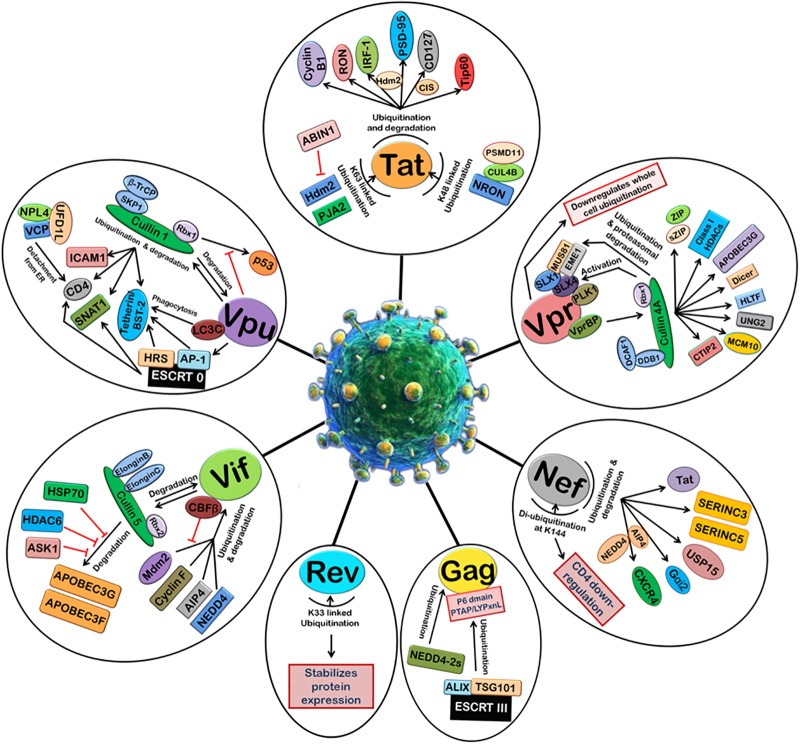
Manipulation of host 26S proteasomal machinery by HIV-1 proteins. HIV-1 proteins redirect the host cellular 26S proteasomal machinery in order to counteract host restriction factors and promote viral replication. Tat induces the ubiquitination and degradation of Cyclin B1, RON, IRF-1 (Hdm2 E3 ligase), PSD-95 (Hdm2 E3 ligase), CD127 (CIS E3 ligase), and Tip60. Tat also undergoes K48-linked polyubiquitination by NRON-CUL4B-PSMD11 complex and K63-linked ubiquitination by Hdm2 and PJA2. ABIN1 inhibits Hdm2-mediated K63-linked ubiquitination of Tat. Vpr can downregulate whole cell ubiquitination and redirect it to degradation of selected host proteins. Vpr binds VprBP-Cullin4A-DCAF-1-DDB-1-Rbx1 E3 ligase complex and causes ubiquitin-dependent degradation of CTIP2, MCM10, UNG2, HLTF, Dicer, APOBEC3G, Class I HDACS, ZIP, sZIP, MUS81, and EME1. Nef induces ubiquitination and degradation of Tat, SERINC3, SERINC5, USP15, Gαi2 (AIP4 E3 ligase), and CXCR4 (AIP4 and NEDD4 E3 ligases). Nef is de-ubiquitinated itself at K144 and causes CD4 downregulation. HIV-1 Gag is also ubiquitinated by ESCRT III machinery and NEDD4-2s which is required for the assembly and release of new viral particles. K33-linked ubiquitination of Rev stabilizes its expression. Vif binds Cullin5-ElonginB-ElonginC-Rbx2 E3 ligase complex to ubiquitinate and degrades APOBEC3G and APOBEC3F. This is inhibited by HSP70, HDAC6, and ASK1. Vif is itself ubiquitinated and degraded by Cullin5-ElonginB-ElonginC-Rbx2 E3 ligase complex and also other E3 ligases such as Mdm2, Cyclin F, AIP4, and NEDD4. CBFβ inhibits Mdm2-mediated ubiquitination of Vif. Vpu induces degradation of ICAM1, CD4, SNAT1, and Tetherin/BST-2 with the help of Cullin1-βTrCP-SKP1-Rbx1 E3 ligase complex and ESCRT 0 complex. Vpu is also ubiquitinated by Cullin1-βTrCP-SKP1-Rbx1 E3 ligase complex. The degradation of p53 by Cullin1-βTrCP-SKP1-Rbx1 E3 ligase complex is also inhibited by Vpu.

Viruses are well-known to manipulate host proteasomal machinery in several ways. Entry or release of different viruses, replication, and many other steps in the life cycle of viruses have been shown to be dependent on the proteasomal machinery (Banks et al., 2003). As a basic immune response, the UPS can be used by the host cell to degrade the invading viral proteins, thereby limiting the viral infection. On the other hand, the same machinery, if hijacked by a virus, is exploited by the virus for its own advantage. Thus, the cellular UPS plays a pivotal role in viral restriction and counter-restriction game (Seissler et al., 2017). Generally, viruses manipulate protein ubiquitination machinery to overcome the host cell defense mechanisms, such as apoptosis, type 1 interferon (IFN) response, and major histocompatibility complex (MHC) class 1 antigen presentation (Figure 2). Many viruses encode one or more proteins that redirect cellular E3s of the UPS to target host antiviral proteins such as p53 (induction of apoptosis) (Verma et al., 2011) and STAT1 (IFN response), while on the other hand some viruses express their own E3 ligases, which can lead to the degradation of cellular defense proteins, such as p53 or MHC class 1 molecules. Such viral strategies were reviewed recently (Gao and Luo, 2006).

HIV-1 is one of the most dreaded viruses which has widely spread throughout the world due to its ability to alter the host machinery to escape the immune response and facilitate its own survival and replication. HIV-1 infects the cells of the human immune system such as helper T-cells (specifically CD4+ T cells), macrophages, and dendritic cells (Cunningham et al., 2010). HIV-1 infection decreases the levels of helper T-cells through a number of mechanisms, such as apoptosis of uninfected bystander cells (Garg et al., 2012), direct viral killing of infected cells, and killing of infected CD4+ T cells by CD8+ cytotoxic lymphocytes that recognize infected cells (Kumar et al., 2012), and causes acquired immunodeficiency syndrome (AIDS). HIV-1 is a lentivirus and belongs to *retroviridae* family which is characterized by the presence of a conical capsid comprising of viral protein p24 (Montagnier, 1999) and reverse transcriptase enzyme. HIV-1 consists of two copies of single-stranded RNA which is 9749 nucleotides long (Ratner et al., 1985; Wain-Hobson et al., 1985). RNA genome of HIV-1 contains LTR (long terminal repeat) which serves as the binding site for host transcription factors, elements of the transcriptional machinery, and virus-encoded proteins that regulate LTR activity and subsequent expression of viral RNA and proteins (Jones and Peterlin, 1994). HIV-1 encodes for three structural proteins, namely Gag, Pol, and Env that are found in all retroviruses, and six non-structural proteins, namely Tat, Rev, Vpr, Vif, Vpu, and Nef that are unique to HIV-1.

The life cycle of HIV-1 is similar to that of other retroviruses. It starts with the attachment of the virus to the specific target cell followed by the viral capsid-containing RNA genome entering the cytoplasm. Viral RNA is reverse transcribed into double stranded DNA with the help of viral reverse transcriptase. Double-stranded proviral DNA is integrated into the host chromosomal DNA, transcribed, and translated into viral proteins. This is followed by the assembly of viral particles and budding of mature virions from the surface of the host cell. HIV-1 Tat and Rev play an early and essential regulatory role in the life cycle of HIV-1. Tat interacts with the TAR (trans-activating responsive) element in LTR promoter (Rana and Jeang, 1999) and increases the steady-state levels of all the viral transcripts. Rev, on the other hand, is mainly involved in the transport of singly spliced or unspliced HIV-1 genomic RNA via a *cis*-acting element called Rev response element (RRE) (Malim et al., 1989b; Hope et al., 1990; Kjems et al., 1991). Other accessory proteins of HIV-1, namely Vif, Vpr, Vpu, and Nef, seem to be dispensable for viral life cycle but have been found to play an important role in the pathogenesis of virus in the host. The detailed mechanisms of their general pathogenesis have been reviewed elsewhere (Sharma and Bhattacharya, 2009; Swanstrom and Coffin, 2012).

Due to the small size of HIV-1 genome, it encodes few proteins and relies on the host cellular machinery for other functions (Brass et al., 2008; Bushman et al., 2009; Swanstrom and Coffin, 2012). HIV-1 proteins play important roles in viral pathogenesis by exploiting the host machinery to evade the anti-viral immune responses (Friedrich et al., 2011; Zheng et al., 2012). The most common function of these proteins is to manipulate the host ubiquitin-dependent proteasomal degradation machinery by exploiting multiple pathways. HIV-1 proteins alter the specificity of cellular E3 ligases in a manner that helps viral replication, for example, UPS has been found to be vital for the NF-κB signaling (Strebel, 2013), which is known to be utilized by HIV-1, and the final assembly and release of viral particles from infected cells (Alroy et al., 2005; Iwai, 2010). The nuclear factor NF-κB pathway is a well-known proinflammatory signaling pathway (Lawrence, 2009). It plays a pivotal role in the expression of proinflammatory genes such as cytokines, chemokines, and adhesion molecules (Lawrence, 2009). HIV-1 LTR is regulated by its own viral proteins as well as host factors such as NF-κB that becomes activated during a virus infection (Stroud et al., 2009). HIV-1 genome contains two tandem repeats in its LTR region, which are the binding sites of NF-κB and are among the most highly conserved sequences of HIV-1 genome (Stroud et al., 2009; Bachu et al., 2012). It has helped HIV-1 evolution in the great deal (Bachu et al., 2012). HIV-1 Env cytoplasmic domain has been recently reported to activate NF-κB pathway (Beraud et al., 2018). Contrary to that, HIV-1 protein, Vpu was reported to cause inhibition of viral replication through modulating NF-κB signaling pathway and thereby subsequent immune responses (Bour et al., 2001). Other examples of such processes are found in the mechanisms adopted by the virus to egress from infected cells, which will be explained later.

Similarly, cell cycle progression is typically regulated by the sequential degradation of multiple cell cycle proteins, and viral replication is highest in G2/M-arrested stage (Groschel and Bushman, 2005). HIV-1 replication is reported to be affected by a number of anti-viral factors including ISG15 (Interferon stimulated gene 15) and TRIM5α (Tripartite motif-containing protein 5 alpha). These anti-viral factors interact with the incoming HIV-1 virion particle (Zhao et al., 2011). TRIM proteins play an extremely important role in the innate immune defense, especially against retroviral infections such as HIV-1 (Rold and Aiken, 2008). Rhesus macaque TRIM5α targets the HIV-1 capsid and disrupts the infection phenomenon at an early post-entry stage, just before the reverse transcription (Rold and Aiken, 2008). TRIM5α has also been reported to interact with proteasomal complex. Forster resonance energy transfer (FRET) analysis indicated that there is a direct interaction between TRIM5 and PSMC2 (part of proteasomal complex). Fluorescence microscopy also revealed co-localization of proteasomes and TRIM5α cytoplasmic bodies (Diaz-Griffero et al., 2006). Similarly, ISG15 is a type I interferon-induced ubiquitin-like modifier (Morales and Lenschow, 2013). ISG15, a small protein of just 15 kDa, has the potential to conjugate with 100s of target proteins. ISG15 is found in both forms—an unconjugated form inside the cell and a freely circulating released form which gets secreted from interferon-stimulated cells into the extracellular environment (Morales and Lenschow, 2013). Conjugation of ISG15 with its target protein is popularly referred to as ISGylation process. The processes of ISGylation and general protein ubiquitination share a lot of similarities and both of these protein modifiers regulate a myriad of biological processes (Harty et al., 2009). HIV-1 is known to be inhibited by ISG15. IFNs are in general known to inhibit retroviral and lentiviral virion release; ISG15 has specifically been reported to be involved in IFN-mediated inhibition of HIV-1 (Kunzi and Pitha, 1996; Kunzi and Pitha, 2003). Previous studies have indicated that ISG15 disrupts the early stages in the endosomal sorting complexes, required for transport (ESCRT) pathway, in an attempt to hamper HIV-1 budding (Pincetic et al., 2010). The functions and mechanism of anti-viral activities of ISG15 have been extensively reviewed elsewhere (Villarroya-Beltri et al., 2017; Perng and Lenschow, 2018). Hence, the UPS influences multiple interactions between HIV-1 proteins and various cellular Ub ligase complexes, which are discussed in this review.

##### HIV-1 Tat

HIV-1 Tat is one of the two essential viral regulatory factors (Tat and Rev) for HIV-1 gene expression. HIV-1 Tat has been a key focus of HIV research since its discovery in 1985 (Sodroski et al., 1985; Lawrence, 2009). It plays a crucial role in activating viral gene transcription and performs several other functions which have significant implications for viral pathogenesis. Tat is a small protein of 9–11 kDa, consisting of 86 to 101 amino acids depending on the subtype (Jeang, 1996). It acts by binding to the TAR RNA element and activating transcription initiation and elongation from the LTR promoter. Transcriptional activity of Tat has been found to be stimulated by ubiquitination. Hdm2 (human homolog of Mdm2, an E3 Ubiquitin ligase) is the positive regulator of transcriptional activity of Tat and has been found to mediate its ubiquitination (Brès et al., 2003). Here, instead of Lys-48 (involved in targeting the substrate for proteasomal degradation), Lys-63-linked polyubiquitin chains have been found to be involved in ubiquitination of Tat which is believed to play a regulatory function and does not lead to proteolysis (Pickart, 2000). Our group has recently reported that HIV-1 Tat stabilizes Mdm2 (mouse double minute 2 homolog) by inducing its phosphorylation at S166 through AKT (Raja et al., 2017), thus creating a positive feedback loop between Tat and Mdm2. Lys-63-linked polyubiquitination of Tat has recently been reported to be inhibited by ABIN1 (A-20 binding inhibitor of NF-κB activation) via redistribution of Hdm2 from nucleus to cytoplasm (Chen et al., 2017). A recent report suggests that PJA2, a RING Finger E3 ligase, ubiquitinates HIV-1 Tat in a non-degradative manner and thus regulates the transcriptional activity of Tat. Polyubiquitination of Tat by PJA2 requires the presence of Tat in P-TEFb complex (Faust et al., 2017).

HIV-1 Tat is also reported to induce Hdm2-mediated ubiquitination and proteasomal degradation of IRF1 (Interferon Regulatory Factor 1), thereby quenching the transcriptional activity of IRF1 and evading host immune responses (Remoli et al., 2016). Tat-induced ubiquitination has been recently found to play an important role in HIV-associated neurocognitive disorder (HAND), characterized by impaired *N*-methyl-D-aspartate receptors (NMDARs), via GluN2A/Akt/Mdm2 pathway (Green and Thayer, 2016). NMDARs control synaptic plasticity and memory function (Li and Tsien, 2009). Tat binds and potentiates NMDARs, but they adapt in the continued presence of Tat. This is because potentiated NMDAR activates AKT kinase which further activates Mdm2, an E3 ubiquitin ligase. Mdm2 ubiquitinates and degrades PSD-95, which is a scaffold protein, and forms a complex with NMDARs and regulates their surface expression, localization, and coupling to signaling pathways (Kim and Sheng, 2004; Chen et al., 2015). GluN2A-containing NMDARs are essential for Tat-induced adaptation of NMDAR activity. This leads to synaptic loss that results in cognitive decline in HAND (Green and Thayer, 2016).

Recently, Tat has also been found to bind CD127 and recruit CIS (E3 ligase) to the cytoplasmic tail of CD127, even in the absence of IL-7 and receptor phosphorylation, thereby inducing ubiquitination and proteasomal degradation of the receptor. This decreases IL-7 signaling and adversely affects the survival and functioning of CD8 T-cells (Sugden et al., 2016). Tat is also known to induce proteasomal degradation of the receptor tyrosine kinase RON, which is a regulator of inflammation, facilitating the evasion of host immune responses and promoting viral replication (Kalantari et al., 2008). Tat also interacts with N-terminus of Cyclin B1 and stimulates its polyubiquitination and proteasomal degradation (Zhang et al., 2010), thus keeping the expression of Cyclin B1 (which is induced by Tat itself at the transcriptional level) under control to probably inhibit apoptosis and facilitate viral replication. Tat has also been observed to induce the polyubiquitination and proteasomal degradation of Tip60 by p300/CBP-associated E4-type ubiquitin-ligase activity in order to inhibit the cell apoptosis caused by Tip60 in response to DNA damage, and ensures survival of the virus (Col et al., 2005).

Tat has also been found to undergo Lys-48-linked ubiquitination followed by its proteasomal degradation (Zhang et al., 2014). It is well-known that HIV-1 latency can be due to deficiency of Tat protein (Kao et al., 1987). A recent study reported that NRON, a long non-coding RNA (lncRNA), plays a key role in maintaining HIV-1 latency by inducing specific degradation of Tat (Li et al., 2016). Here, NRON has been shown to directly link Tat with ubiquitin ligase complex containing CUL4B and PSMD11, leading to its proteasomal degradation.

##### HIV-1 Rev

Rev is the second most crucial regulatory factor for HIV-1 gene expression. Rev is a small 19-kDa phosphoprotein, localized mainly in the nucleolus/nucleus, and it is expressed during the early stages of HIV-1 life cycle (Cochrane et al., 1989; Malim et al., 1989a). It cycles rapidly between the nucleus and the cytoplasm. The characteristic function of Rev is to export HIV-1 mRNA that contains a RRE from the nucleus to the cytoplasm to facilitate the production of structural proteins of the virus. In the absence of Rev, the host RNA splicing machinery quickly splices the RNA so that only the regulatory proteins, Rev and Tat, and the accessory protein Nef can be produced. HIV-1 Rev has not been reported to induce ubiquitination and proteasomal degradation of any host protein till now. Also, degradative polyubiquitination of Rev is not yet reported. However, there is one report showing that Rev is modified by Lys-33-linked polyubiquitination which increases the abundance of the protein rather than decreasing the stability (Vitte et al., 2006). Also, impairment of its ubiquitination decreases the stability but does not affect its functional activity (Vitte et al., 2006).

##### HIV-1 Nef

Nef is an accessory protein of the HIV-1 and one of the earliest HIV proteins to be produced in infected cells. It is a multifunctional myristoylated protein of 27 kDa size. Nef is predominantly found in the cytoplasm and is associated with the plasma membrane (Niederman et al., 1993) via the myristoyl residue linked to the conserved second amino acid residue (Gly). It is well-known to increase the infectivity of the virus (Chowers et al., 1994). Nef has also been observed to redirect the host ubiquitination machinery to benefit the virus. It downregulates the expression of CD4, primary receptors used by HIV-1 for infection of helper T cells, thus preventing the superinfection and premature death of the infected cells facilitating the efficient viral replication. It involves Nef-mediated endocytosis of CD4 receptor which occurs by the de-ubiquitination of Nef at Lys-144. Mutation at lysine 144 position of Nef protein completely abrogates its ability to downregulate CD4 receptors (Jin et al., 2008). HIV-1 Nef also downregulates CXCR4 by inducing its ubiquitination via an E3 ligase AIP4 (**itchy E3 ubiquitin protein ligase)** or NEDD4 (Neural precursor cell expressed, developmentally downregulated 4), but directs it to lysosomal degradation (Chandrasekaran et al., 2014). Nef also impairs heterotrimeric G-protein signaling by inducing AIP4 E3 ligase-mediated ubiquitination of Gαi2 followed by its lysosomal degradation which impairs leukocyte trafficking leading to immune dysfunction in the early phase of HIV-1 infection (Chandrasekaran et al., 2012). HIV-1 Nef is also observed to cause a noticeable decay of USP15, in a ubiquitin-dependent manner. Conversely, USP15 also decreases the expression of Nef by ubiquitination and proteasomal degradation (Pyeon et al., 2016). Nef also increases the infectivity of the virus by inducing proteasomal degradation of multipass transmembrane proteins, serine incorporator 3 (SERINC3) and SERINC5, thus preventing their incorporation into HIV-1 virions (Matheson et al., 2015; Usami et al., 2015; Trautz et al., 2016). Nef is also reported to induce cytoplasmic localization of Tat and mediate its degradation via ubiquitin-dependent proteasomal pathway to optimize the viral replication (Sugiyama et al., 2011a).

##### HIV-1 Vif

Vif is a basic HIV-1 protein of 23 kDa. Vif has been reported to be essential for efficient viral replication, thus producing infectious viral progeny (Gabuzda et al., 1992; Von Schwedler et al., 1993; Kao et al., 2003). In the absence of Vif, the defective viral particles are produced. Vif is a cytoplasmic protein, existing in both a soluble cytosolic form and a membrane-associated form. It also promotes the infectivity of the virus like Nef does but not the production of viral particles. To increase the infectivity of the virus, Vif counteracts an important cellular restriction factor, APOBEC-3G (Apolipoprotein B mRNA-editing enzyme catalytic polypeptide like 3G), as well as other members of APOBEC3 family.

In the absence of Vif, APOBEC3G is packed into the viral particles and gets transferred to the newly infected neighboring cells where it induces hypermutation of the single-stranded proviral cDNA by causing deamination of Cytosine residues to Uracil residues. These mutations can be recognized by UNG2 (Uracyl *N*-Glycosylase 2) which degrades proviral DNA by endonuclease activity (Yang et al., 2007). Vif prevents this hypermutation by binding and inducing polyubiquitination of APOBEC3G followed by its proteasomal degradation, thus inhibiting the incorporation of APOBEC3G into HIV-1 virions (Conticello et al., 2003; Shao et al., 2010). For this, Vif binds directly with Cullin-RING ubiquitin ligase (CRL) complex (Cullin5, Elongin B, and Elongin C (Vif-BC-Cul5) through a novel SOCS-box motif, which then mediate cullin-5 (CUL5)–ring-box-1 (RBX1)-dependent ligation of ubiquitin (Ub) to APOBEC3G. The ability of Vif to suppress antiviral activity of APOBEC3G is specifically dependent on this complex, which allows Vif to interact with APOBEC3G and induce its ubiquitination and degradation (Yu et al., 2003; Kobayashi et al., 2005). SOCS-box motif of HIV-1 Vif is found to be involved in the formation of Cul5-EloBC complex and Vif-mediated degradation of APOBEC3G (Mehle et al., 2004). HIV-1 Vif is also reported to induce polyubiquitination and proteasomal degradation of another human cytidine deaminase APOBEC3F, a protein related to previously described host restriction factor APOBEC3G, by Vif-Cul5-ElonB/C E3 ubiquitin ligase complex (Liu et al., 2005).

Heat shock protein 70 (HSP70) has been found to inhibit ubiquitination and proteasomal degradation of APOBEC3G by Vif via disruption of interaction between Vif and APOBEC3G. Therefore, siRNA-mediated knock-down of HSP70 in cells enhances the Vif-mediated degradation of APOBEC3G (Sugiyama et al., 2011b). Recently, HDAC6 has been identified as a new antiviral factor which forms a complex with APOBEC3G and protects it from Vif-mediated ubiquitination and proteasomal degradation via promoting autophagic degradation of Vif (Valera et al., 2015). Another host protein ASK1 (apoptosis signal-regulating kinase 1) has recently been found to interfere with Vif-mediated ubiquitination and degradation of APOBEC3G by binding to BC-box of Vif and disrupting the assembly of Vif-Cul5 E3 ligase complex. Interestingly, expression of ASK1 is induced by antiretroviral drug AZT (zidovudine) treatment (Miyakawa et al., 2015).

Vif is also ubiquitinated and degraded (Fujita et al., 2004) by cellular proteins as a part of host innate anti-viral response. Mdm2, an E3-ubiquitin ligase, decreases the expression of HIV-1 Vif by inducing its polyubiquitination and subsequent proteasomal degradation (Izumi et al., 2009). A host transcription co-factor, core binding factor β (CBFβ), has been recently reported to protect Vif from Mdm2-mediated proteasomal degradation by binding with Vif and inhibiting its interaction with Mdm2 (Matsui et al., 2016). CBFβ is also known to be required for assembly of Vif-Cul5 E3 ubiquitin ligase complex and proteasomal degradation of APOBEC3G (Jäger et al., 2011; Zhang et al., 2011). Cyclin F has been recently identified as a novel host restriction factor which binds with Vif and induces its polyubiquitination by SCF-cyclin F E3 Ligase complex followed by its proteasomal degradation (Augustine et al., 2017). Vif has also been found to be post-translationally modified by two HECT (Homologous to the E6-AP Carboxyl Terminus) E3 ubiquitin ligases, AIP4 and NEDD4-mediated ubiquitination (Dussart et al., 2004). Vif has also been reported to undergo auto-ubiquitination by Cul5 E3 ligase complex (Mehle et al., 2004), which is required for polyubiquitination and subsequent degradation of APOBEC3G (Dang et al., 2008).

##### HIV-1 Vpr

Vpr is a 96-amino acid (14 kDa) protein, which is incorporated into the virion (Cohen et al., 1990). It is mainly localized to the nucleus and its localization is controlled by its leucine-isoleucine rich (LR) motif (Mahalingam et al., 1997). The characteristic functions of Vpr are nuclear translocation of the HIV-1 pre-integration complex (PIC) in non-dividing cells by interacting with the nuclear transport pathway (Heinzinger et al., 1994; Fouchier et al., 1998; Nie et al., 1998; Popov et al., 1998), induction of cell cycle arrest at the G2/M phase (He et al., 1995; Jowett et al., 1995) through its C-terminal domain (Mahalingam et al., 1997), and apoptosis of infected cells (Stewart et al., 1997). Vpr also upregulates HIV replication as a result of its cell cycle-modulating activity (Goh et al., 1998; Subbramanian et al., 1998). Vpr protein has been found to synergistically enhance the transcriptional activity of HIV-1 LTR by structural and functional interaction with Tat (Sawaya et al., 2000).

For induction of G2/M cell cycle arrest, Vpr is well-known to recruit cullin4A-DDB1-DCAF1 E3 ubiquitin ligase complex by interacting directly with DCAF1 (DDB1 and CUL4 Associated Factor 1). However, the exact target of this complex is still unknown (Belzile et al., 2007; DeHart et al., 2007; Hrecka et al., 2007; Schröfelbauer et al., 2007; Tan et al., 2007; Wen et al., 2007). It is also reported that Vpr-mediated G2/M arrest involves the activation of the structure-specific endonuclease (SSE) regulator SLX4 complex by Vpr (Berger et al., 2015), which requires VprBP-DDB1-CUL4 E3-ligase complex. Direct interaction between Vpr and SLX4 induces the recruitment of VprBP (Vpr-binding protein) and kinase-active PLK1 (Polo-like Kinase-1). This increases the cleavage of DNA by SLX4-associated MUS81 (Crossover junction endonuclease) and EME1 (Essential Meiotic Structure-Specific Endonuclease 1) endonucleases, which results in cell cycle arrest at G2/M phase. SLX4 activation also helps HIV-1 to escape the host anti-viral innate immune responses (Laguette et al., 2014). Vpr also binds the DNA replication factor MCM10 (Mini Chromosome Maintenance 10) in a VprBP-dependent manner and induces proteasomal degradation of MCM10 using cullin4A-DDB1-DCAF1 E3 ligase activity to induce G2/M cell cycle arrest (Romani et al., 2015).

Vpr also exploits cullin4A-DDB1-DCAF1 E3 ligase complex to interfere with DNA damage response in host cells to support viral replication. Vpr interferes with base excision repair pathway by inducing ubiquitination and proteasomal degradation of DNA glycosylase UNG2, thus protecting the virus from the action of host restriction factor APOBEC3G (Schröfelbauer et al., 2005; Ahn et al., 2010; Wu et al., 2016). HIV-1 Vpr is also reported to directly induce the proteasomal degradation of APOBEC3G using the same cullin4A-DDB1-DCAF1 E3 ligase complex which is mediated by VprBP (Zhou et al., 2015). Vpr has also been found to induce the proteasomal degradation of DNA translocase HLTF (Helicase-Like Transcription Factor) involved in the repair of damaged replication forks using cullin4A-DDB1-DCAF1 E3 ligase complex in T cells and macrophages by directly interacting with DNA-binding residues in the N-terminal HIRAN (HIP116 Rad5p N-terminal) domain of HLTF. This function of Vpr is independent of G2/M cell cycle arrest (Lahouassa et al., 2016; Zhou et al., 2017). SLX4, which is activated by Vpr, is also reported to bind with the catalytic SLX1 through c-terminus to form an SLX4-SLX1 complex. The SLX4-SLX1complex further associates with MUS81-EME1 endonucleases, which are involved in homologous recombination and replication fork processing and repair (Ciccia et al., 2003; Gaillard et al., 2003; Hanada et al., 2006), forming a stable holoenzyme termed SLX4com. Vpr is recently reported to target this four-subunit complex, SLX4-SLX1-MUS81-EME1, ultimately leading to the ubiquitination and proteasomal degradation of MUS81 and EME1 (Zhou et al., 2016).

It has been observed that proteasomal degradation of the endoribonuclease Dicer, one of the most important proteins of host microRNA machinery, is also induced by Vpr using cullin4A-DDB1-DCAF1 E3 ubiquitin ligase complex, thus leading to the enhanced HIV-1 replication in macrophages (Casey Klockow et al., 2013). Vpr is also involved in the enhanced turnover of CTIP2 (COUP-TF-interacting protein 2) in microglial cells via cullin4A-DDB1-VprBP-DCAF1 E3 ubiquitin ligase, thus counteracting CTIP2-mediated silencing of HIV-1 gene transcription to favor viral replication. Vpr is also known to induce cullin4A-DDB1-DCAF1 E3 ubiquitin ligase-mediated degradation of two transcriptional regulators ZIP (leucine Zipper) and sZIP, adaptors of NuRD (Nucleosome Remodeling Deacetylase) chromatin remodeling complex (Maudet et al., 2013). The class I HDACs (Histone Deacetylase 1, 2, 3, and 8) are also degraded by Vpr via cullin4A-DDB1-VprBP-DCAF1 E3 ubiquitin ligase activity which helps HIV-1 to overcome latency in macrophages (Romani et al., 2016). Our lab had recently reported that HIV-1 Vpr downregulates the whole cell ubiquitination in infected cells; however, the ubiquitination machinery was redirected to degrade specific anti-viral restriction factors (Arora et al., 2014). This suggests that Vpr redirects the host proteasomal machinery, so as to accelerate the degradation of anti-viral restriction factors, and promotes viral replication.

##### HIV-1 Gag

The Gag precursor of HIV-1 possesses matrix (MA, p17) at the N-terminus, capsid (CA, p24), nucleocapsid (NC, p7), and a C-terminal domain called p6 that is unique to primate lentiviruses, as well as two “spacer” regions which separate CA from NC, and NC from p6 (Mervis et al., 1988; Henderson et al., 1992). The 55 kDa Gag precursor is also known as assemblin to indicate its role in viral assembly. The HIV-1 Gag precursor is synthesized on cytosolic ribosomes (Tritel and Resh, 2000) and becomes co-translationally modified by the N-terminal attachment of a myristyl group, which increases its affinity for membranes (Göttlinger et al., 1989; Bryant and Ratner, 1990). Gag oligomerizes underneath the plasma membrane through protein–protein and protein–RNA interactions, with the MA domain oriented toward the lipid bilayer which helps in the targeting of Gag to the plasma membrane and in the incorporation of the viral envelope (Env) glycoproteins into nascent particles.

There are several lines of evidence suggesting that ubiquitination of Gag plays an important role in the assembly of new viral particles (Gottwein and Krausslich, 2005). HIV-1 virus release depends on the ESCRT (endosomal sorting complexes required for transport) machinery (Morita et al., 2011). ESCRTs recognize and process ubiquitinated proteins. HIV-1 Gag polyprotein gets ubiquitinated at its p6 domain for its interaction with the ESCRT machinery (Gottlinger et al., 1991; Huang et al., 1995; Sette et al., 2013). The p6 domain of HIV-1 Gag possesses two types of L domains. The L domain containing PTAP motif is required for its interaction with the host ubiquitination machinery (Vogt, 2000; Martin-Serrano et al., 2001; VerPlank et al., 2001). The second L domain of Gag is of the LYPxnL-type and binds to the V domain of ALIX (apoptosis-linked gene 2-interacting protein X)/AIP1 (ALG-2 interacting protein 1) (Martin-Serrano et al., 2003; Strack et al., 2003; von Schwedler et al., 2003; Fisher et al., 2007; Zhai et al., 2008), which interacts with the components of ESCRT III (Katoh et al., 2004; Pires et al., 2009). Tsg101 (Tumor susceptibility gene 101), an integral part of ESCRT-I and a homolog of ubiquitin-conjugating (E2) enzymes, forms a ternary complex with AIP1 and HIV-1 Gag and facilitates viral budding through specific isoforms of ESCRT-III, with the CHMP2 (Charged Multivesicular body Protein 2) and CHMP4 (Charged Multivesicular body Protein 4) families playing key functional roles (Garrus et al., 2001; VerPlank et al., 2001; von Schwedler et al., 2003; Morita et al., 2011). Membrane flotation experiments revealed that ubiquitinated Gag is highly enriched in membrane-bound fractions, thereby suggesting that the association of Gag with cell membrane is essential for its ubiquitination (Kim and Sheng, 2004; Jager et al., 2007).

NEDD4-type ubiquitin ligases also stimulate HIV-1 budding by inducing ubiquitination of Gag. PPxY-type L domains constitute ligands for NEDD4-type ubiquitin ligases. Despite the absence of PPxY motif in HIV-1 Gag, the release of HIV-1 particles is potently enhanced by ectopic NEDD4-2s, a native isoform with a naturally truncated C2 domain which acts as an autonomous Gag-targeting module and can be replaced by HIV- 1 Gag-binding protein, cyclophilin A (CypA) (Weiss et al., 2010). Mono-ubiquitination of lysine residues (K27 and K33) of the p6 domain and further cumulative ubiquitination of NC-p2 (NucleoCapsid-peptide 2) domain are required for budding of new virions (Ott et al., 1998). Also, lysine residues in MA, CA, NC, p6, and SP2 domains are mono or di-ubiquitinated at almost similar levels compared to the other domains. Deletion of all lysine residues in just one domain had minor effects on virus release. However, cumulative mutations in multiple domains, such as NC and SP2 or in NC and p6, resulted in an accumulation of late viral budding particles. Using a similar technique, they also showed that ubiquitination of downstream of CA domain causes significant reduction in virus release kinetics (Gottwein and Krausslich, 2005). Overall, these results suggest that ubiquitination of lysine residues in Gag near viral late domain is crucial for HIV-1 budding. This also makes it easy to comprehend why Gag ubiquitination is critically involved in sequential protein interaction network at the budding site.

##### HIV-1 Vpu

Vpu, one of the auxiliary proteins, is a 16 kDa (81 amino acids) type I integral transmembrane protein which is co-translationally inserted into the membranes of infected cells (Strebel et al., 1989). Vpu has two primary biological functions: **(a)** It enhances virion release from the plasma membrane of HIV-1-infected cells. If Vpu is deleted from the viral genome, newly formed viral particles remain tethered to the outer surface of the plasma membrane after budding and eventually get endocytosed and digested (Neil et al., 2006); **(b)** It downregulates the expression of CD4 at the surface of helper T-cells.

The cellular restriction factor Tetherin or BST-2 (Bone marrow Stromal cell Antigen 2) is mainly responsible for the blocking of virus release. BST-2 is a disulfide-bond-linked dimer which stays anchored or embedded in the plasma membrane by two domains: a transmembrane domain close to its N-terminus and an extracellular C-terminal glycosyl-phosphatidylinositol (GPI)-anchor. These two domains mediate virion-tethering to the host cell, one remaining in the plasma membrane and the other inserted into the viral envelope (Hinz et al., 2010). BST-2 life span and stability are mainly regulated by ubiquitination and lysosomal degradation. It is mediated by the cellular E3 ubiquitin ligases MARCH8 and NEDD4. However, Vpu does not affect the constitutive cellular ubiquitylation machinery of BST-2 (Roy et al., 2017). It counteracts BST-2 via direct binding with transmembrane domains of BST2 and directs it to beta TrCP (Beta-transducin repeats-containing protein)-mediated proteasomal degradation via cullin1-Skp1 E3 ligase complex (Mangeat et al., 2009). There are also few reports stating that Vpu-induced beta TrCP-mediated degradation of BST-2 is reduced by lysosomal inhibitors but not MG132, a proteasomal inhibitor (Douglas et al., 2009; Gustin et al., 2012). The cytoplasmic DSGxxS motif of Vpu is required for interaction with the E3-ubiquitin ligase adaptor, β-TrCP (Margottin et al., 1998; Coadou et al., 2003; Douglas et al., 2009). It was also shown that almost all the potential ubiquitination sites (lysines, cysteines, serines, and threonines) within the cytoplasmic domain of BST-2 contributed to its ubiquitination and degradation by Vpu. However, ubiquitination at serine-threonine-serine sequence was responsible for mediating the downregulation of BST-2 from the cell surface (Tokarev et al., 2011). It has been observed that Vpu may ubiquitinate BST-2 by targeting N-terminus of BST-2 for ubiquitination even when its lysine, serine, and threonine residues in cytoplasmic domains are mutated (Strack et al., 2003). Vpu itself is also polyubiquitinated and degraded by SCF E3 ligase complex in a beta-TrCP-dependent manner (Belaïdouni et al., 2007; Estrabaud et al., 2007). Phosphorylation of Vpu at S61, which lies adjacent to beta-TrCP binding motif, induces its degradation in a beta-TrCP-independent manner (Estrabaud et al., 2007).

HIV-1 Vpu can downregulate the surface levels of BST-2 by other mechanisms also, even without the involvement of betaTrCP (Mitchell et al., 2009; Tervo et al., 2011; Ramirez et al., 2015). Vpu has recently been reported to bind BST-2 and target it to ESCRT-dependent endosomal degradation via interaction with clathrin adaptor AP-1. In this study, it was shown that phosphorylation of Vpu is required for this process (Kueck et al., 2015; Pujol et al., 2016). HRS (hepatocyte growth factor-regulated tyrosine kinase substrate), a key component of ESCRT 0 complex, has also been shown to promote the release of HIV-1 virions from the surface of infected cells by Vpu-mediated ESCRT-dependent sorting for lysosomal degradation of BST-2 (Janvier et al., 2011). Vpu has also been shown to decrease the surface levels of BST-2 by exploiting the autophagy pathway. It can bind with ATG8 (Autophagy related protein 8) ortholog LC3C and induce the removal of BST-2 from HIV-1 budding sites by LC3C-mediated phagocytosis (Madjo et al., 2016). HIV-1 Vpu can also promote virion release from the surface of infected cells even without downregulating BST-2 (Miyagi et al., 2009).

The second important function of HIV-1 Vpu is to downregulate the expression of CD4 which also requires the activity of beta TrCP-cullin1-Skp-1 E3 ligase complex. Vpu binds with beta TrCP through WD repeats in its C-terminus and forms CD4-Vpu-beta TrCP ternary complex. Beta TrCP directs CD4 to polyubiquitination and proteasomal degradation through F-box near its N-terminus which interacts with Skp1p, a targeting factor for ubiquitin-mediated proteolysis by SCF (Skp1–Cullin–F-box) complex (Margottin et al., 1998). Vpu interacts with CD4 in the endoplasmic reticulum. Hence, Vpu-mediated depletion of CD4 also involves Endoplasmic reticulum-associated degradation (ERAD) pathway which is normally used for the turnover of unwanted ER membrane proteins by proteasomal degradation in the cytosol. K-48-linked polyubiquitin chains attached to the cytoplasmic tail of CD4 are recognized by UFD1L (Ubiquitin Fusion Degradation protein 1) which is a part of VCP-UFD1L-NPL4 (Valosin Containing Protein- Ubiquitin Fusion Degradation protein 1- Nuclear Protein Localization protein 4) complex. VCP induces the detachment of CD4 from the ER membrane into the cytosol through its ATPase activity, followed subsequently by proteasomal degradation of CD4. Trp22 in the transmembrane domain of Vpu and Gly415 in the transmembrane domain of CD4 are critically required for Vpu-mediated downregulation of CD4 (Willey et al., 1992; Margottin et al., 1998; Schubert et al., 1998; Binette et al., 2007; Magadán et al., 2010; Magadán and Bonifacino, 2012; Petris et al., 2014).

There are other cellular proteins which are also targeted by Vpu for ubiquitination and proteasomal degradation through beta TrCP and SCF (cullin1-Skp1-F-box) E3 ligase complex. Vpu induces degradation of sodium-coupled neutral amino acid transporter 1 (SNAT1; required for T-cell mitogenesis) through SCF complex in a beta-TrCP-dependent manner, thus interfering with immune-metabolism of the host. Similar to CD4 and BST-2, SNAT1 also undergoes endolysosomal degradation by ESCRT machinery (Matheson et al., 2015). Vpu also downregulates the cell surface glycoprotein ICAM-1 (intercellular adhesion molecule 1) through E3 ligase activity of beta TrCP-SCF complex, thus facilitating the evasion of cell-mediated immunity of the host by preventing the killing of infected CD4+ helper T-cells by NK cells. Transmembrane domain and DSGxxS motif of Vpu are also required for this function (Sugden et al., 2017). Our laboratory has previously reported that Vpu can also suppress the ubiquitination of p53 in a β-TrCP-dependent manner and promote apoptosis during HIV-1 infection (Verma et al., 2011).

##### HIV-1 Proteins and DUBs

**FIGURE 3:**
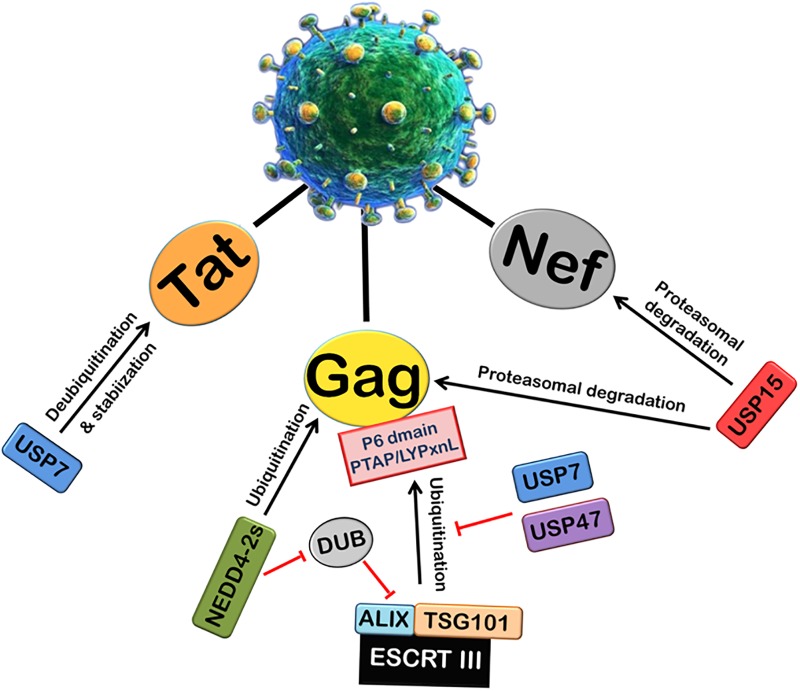
Role of Deubiquitinases (DUBs) in HIV-1 replication. Ubiquitin-Specific Proteases (DUBs) play an important role in viral replication. The ubiquitination and processing of Gag by ESCRT III machinery is inhibited by USP7 and USP47. A deubiquitinase DUB binds ALIX and inhibits the viral release. NEDD4-2s E3 ubiquitin ligase inhibits the action of DUB. USP7 also deubiquitinates and stabilizes HIV-1 Tat protein. In contrast, another deubiquitinase USP15 induces proteasomal degradation of HIV-1 Nef and Gag proteins.

Viruses can also alter the activity of cellular DUBs by encoding proteins which are able to interact with cellular DUBs. Many studies regarding HIV-1 have recently explored the possible role of several members of cellular Ubiquitin-Specific Protease family (USPs) (Figure 3). USP15 has been found to interact with HIV-1 Nef and decrease its expression by ubiquitination and proteasomal degradation. USP15 has also been found to induce the degradation of HIV-1 structural protein Gag via both endosomal and proteasomal degradation pathways, and decrease viral replication (Pyeon et al., 2016). USP7 and USP47s play an intricate and crucial role in HIV-1 replication. By using specific inhibitors (P22077 and PR-619) against USP7 and USP47, respectively, it was shown that USP7 and USP47s regulate Gag processing and modulate the infectivity of the released viral particles. Further, it affects the entry of Gag into the UPS and its display on MHC-I (Setz et al., 2017). In this report, a deubiquitinase was found to be fused with Alix (DUB-Alix), which suppressed the HIV-1 access to TSG101 and blocked the viral release. Interestingly, when co-expressed with NEDD4-2s (ubiquitin ligase), the ability of DUB-TSG101 to diminish HIV-1 release was also reverted. Similarly, incorporation of stably fused Gag-Ub proteins into virus particles made the viral release refractory to DUB-ESCRT-mediated inhibition (Morita et al., 2011). Our lab also recently reported that USP7 deubiquitinates and stabilizes HIV-1 Tat protein, thus leading to the enhancement of HIV-1 replication (Ali et al., 2017).

### Ubiquitin-Independent Proteasomal Degradation Pathway

**FIGURE 4:**
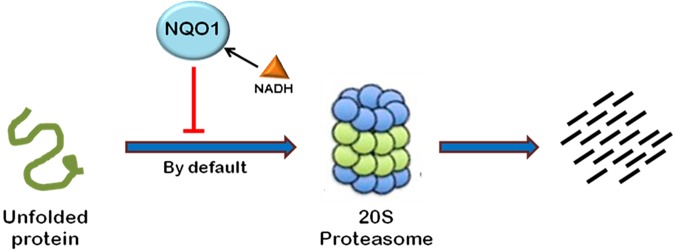
Ubiquitin-independent proteasomal degradation pathway. The proteins containing unstructured regions or unfolded proteins are targeted to 20S proteasome for degradation by default. This pathway is independent of ubiquitination of the protein. Ubiquitin-independent 20S proteasomal degradation of proteins is regulated by NQO1. In the presence of NADH, NQO1 inhibits the degradation of proteins by 20S proteasome.

Most of the cellular proteins are polyubiquitinated and then targeted to 26S proteasome for degradation. However, there are some cases where proteasome plays an important role in post-translational processing of protein. For example, NF-κB is activated by proteasomal processing of p105 into p50 (Rape and Jentsch, 2002). Recently, it has been found that some proteins are also degraded independent of ubiquitination through 20S proteasome by default (Asher et al., 2006). The proteins containing intrinsically unstructured regions have been found to be degraded in a ubiquitin-independent manner (Figure 4). The well-known example of such a protein is the enzyme ornithine decarboxylase (ODC) (Zhang et al., 2003; Asher et al., 2005a). Ubiquitin-independent degradation of p53, a key cell cycle regulator, has also been reported, although p53 is also well-known to undergo ubiquitin-dependent degradation (Asher and Shaul, 2005). This pathway is also reported to promote the degradation of some other cellular proteins such as p73, p21, PGC1-a, and BIMEL that possess many unstructured regions (Asher et al., 2005b; Wiggins et al., 2011; Adamovich et al., 2013). Also, misfolded or highly oxidized proteins are also subjected to ubiquitin-independent degradation by 20S proteasome under conditions of cellular stress (Davies, 2001; Shringarpure et al., 2003). More than 30% of the total cellular proteins possess substantial intrinsic structural disorders which may be targeted for 20S proteasome-mediated degradation (Gsponer et al., 2008; Baugh et al., 2009). Also, 20S proteasomal degradation is a passive process that only needs unstructured regions in the substrate protein (Asher et al., 2006).

20S proteasomal degradation of proteins has been found to be regulated by NADH-bound NAD(P)H quinone oxidoreductase-1 (NQO1) which utilizes NAD(P)H for reduction of various quinones. NQO1 has been named as “gatekeeper” of 20S proteasome. It is physically associated with 20S proteasome. NQO1 has been reported to bind and protect certain proteins, namely P53, p73, and ODC, from 20S proteasomal degradation. This process is facilitated by NADH (Asher et al., 2006; Moscovitz et al., 2012). The competitive removal of NADH from NQO1 with chemical compounds, such as dicoumarol {3,30-methylenebis(4-hydroxy)- 2H-chromen-2-one}, curcumin, and diminutol, activates this pathway, thus enhancing the degradation of unstructured proteins (Asher et al., 2005b, 2006; Moscovitz et al., 2012).

**FIGURE 5:**
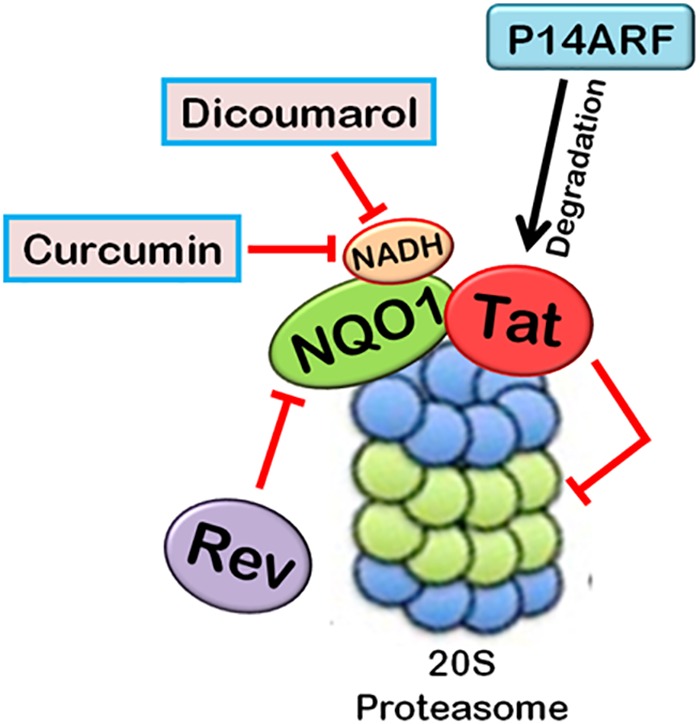
Interplay between HIV-1 proteins and 20S proteasome machinery. 20S proteasomal pathways play a significant role in HIV-1 replication. HIV-1 Tat, being an unstructured protein, is degraded by 20S proteasome. NQO1 inhibits the degradation and stabilizes the expression of Tat in the presence of NADH. HIV-1 Rev downregulates NQO1 and induces the degradation of Tat by 20S proteasome. Dicoumarol and Curcumin are the chemical inhibitors of NQO1 activity. They compete with NADH to bind NQO1, thereby inhibiting its action. P14ARF also induces 20S proteasomal degradation of Tat independent of ubiquitin. HIV-1 Tat, in turn, inhibits the activity of 20S proteasome.

There are a few reports which highlight the role of 20S proteasomal degradation in turnover of HIV-1 proteins (Figures 4, 5). HIV-1 Tat, the principle transactivator of viral LTR promoter, has been found to be a largely unfolded protein. Multiple functions of Tat are governed by its disorderness, which enables it to interact with multiple cellular proteins (Shojania and O’Neil, 2010). Our group has verified the unstructured nature of

Tat protein using multiple algorithms. These programs predicted different degrees of intrinsic disorderness in the Tat protein. The fold index program showed complete disorderness in Tat protein along its entire length with no folded regions at all. Other programs such as RONN, metaPrDOS, and FoldUnfold also predicted substantial unfolded regions in the Tat protein. We reported that Tat is degraded by 20S proteasome and NQO1 protects Tat protein from 20S proteasomal degradation, leading to the stabilization of Tat. Dicoumarol inhibits the action of NQO1 and decreases the expression of Tat. HIV-1 Rev is also found to downregulate the expression of Tat by decreasing the levels of NQO1 (Lata et al., 2015). We subsequently showed that Curcumin, another chemical inhibitor of NQO1, is also reported to induce the degradation of Tat and decrease the viral replication (Ali and Banerjea, 2016). P14ARF has also been found to induce the degradation of HIV-1 Tat via ubiquitin-independent proteasomal pathway (Gargano et al., 2008).

Viruses can also regulate the activity of 20S proteasome. HIV-1 Tat is known to inhibit the peptidase activity of 20S proteasome by interfering with the formation of 20S proteasome-11S regulatory complex, which is involved in antigen processing, thus ensuring the survival of the virus by evading host CTLs’ (Cytotoxic T-lymphocytes) response (Seeger et al., 1997).

## Summary and Future Perspectives

Proteasomal processing is a kind of cellular phenomenon which is required for all the basic cellular maintenance processes. For this reason, viral infections, which are a classic example of total parasitism, depend very heavily on this pathway to redirect cellular resources for their own benefit. HIV-1 proteins, individually and collectively modify the host proteasomal degradation pathway at every step. Each HIV-1 protein directly or indirectly influences the cellular proteasomal degradation machinery to make the cellular environment conducive for optimal virus growth and replication. This is especially true in post-genomic era, where not only the gene-sequences but also their post-transcriptional and post-translational modifications are being reported to have an upper hand in the final outcome of a gene function. A lot of histone–modifications via ubiquitination and de-ubiquitination cycle reveal how little we know about the impact of this crucial protein regulatory mechanism on the gene expression profile. These scenarios highlight the value of this review as we are able to read how each and every HIV-1 proteins has been able to spread by interacting with various components of the UPS system. Here, we have reviewed the various cellular functions of each HIV-1 protein reported so far, which help HIV-1 in exploiting the host proteasomal machinery. This review provides a comprehensive overview of the biology of HIV-1 pathogenesis with a specific perspective of proteasomal machinery. Since ubiquitination and de-ubiquitination have emerged as potential players in pathogenesis, they have provided possible cross points of HIV-1 and host proteasomal machinery. This information can be exploited in future therapeutic approaches against HIV-1 infection.

## Author Contributions

SL designed the study and wrote a majority of the manuscript. RM wrote part of the manuscript. AB assisted with the design of the study and the writing of the manuscript.

## Conflict of Interest Statement

The authors declare that the research was conducted in the absence of any commercial or financial relationships that could be construed as a potential conflict of interest.
